# Combined diffusing capacity for nitric oxide and carbon monoxide as predictor of bronchiolitis obliterans syndrome following lung transplantation

**DOI:** 10.1186/s12931-018-0881-1

**Published:** 2018-09-10

**Authors:** Anna Winkler, Kathrin Kahnert, Jürgen Behr, Claus Neurohr, Nikolaus Kneidinger, Rudolf Hatz, Holger Dressel, Thomas Radtke, Rudolf A. Jörres

**Affiliations:** 1 Institute and Outpatient Clinic for Occupational, Social and Environmental Medicine, Comprehensive Pneumology Center Munich (CPC-M), Ludwig-Maximilians-Universität München, Munich, Germany; 2 Department of Internal Medicine V, University of Munich (LMU), Comprehensive Pneumology Center, Member of the German Center for Lung Research (DZL), Ziemssenstr. 1, 80336 Munich, Germany; 3 Robert-Bosch-Hospital, Klinik Schillerhöhe, Gerlingen, Germany; 4 Department of Thoracic Surgery, University of Munich (LMU), Munich, Germany; 5 Epidemiology, Biostatistics and Prevention Institute, University of Zurich, Zurich, Switzerland; 6 Division of Occupational and Environmental Medicine, University of Zurich and University Hospital Zurich, Zurich, Switzerland

**Keywords:** Lung transplantation, Diffusing capacity, Bronchiolitis obliterans syndrome

## Abstract

**Background:**

There is a need for non-invasive parameters that are sensitive to the development of the bronchiolitis obliterans syndrome (BOS) in lung transplantation (LTx) patients. We studied whether the pulmonary diffusing capacity for inhaled nitric oxide is capable of detecting BOS stages.

**Methods:**

Sixty-one LTx patients were included into this cross-sectional study (19/29/7/3/3 in BOS stages 0/0-p/1/2/3). For analysis stages 0/0-p versus 1/2/3 (“BOS binary-early”), and stages 0/0-p/1 versus 2/3 (“BOS binary-late”) were summarized. Measurements of the combined diffusing capacity for nitric oxide (DLNO) and carbon monoxide (DLCO) were compared with spirometry and bodyplethysmography, and their relative importance was evaluated by discriminant analysis.

**Results:**

Regarding the recognition of “BOS binary-early”, among spirometric parameters forced expiratory volume in 1 s (FEV_1_) was best, among bodyplethysmographic parameters airway resistance, and among diffusing parameters DLNO. Regarding “BOS binary-late”, DLNO was inferior to bodyplethysmographic parameters.

**Conclusion:**

Although the study comprised only measurements at a single time point and no follow-up, DLNO outperformed FEV_1_, the time course of which is used in detecting BOS. Together with its pathophysiological plausibility, this result suggests that the measurement of DLNO, possibly over time, could be an easily applicable tool for the monitoring of LTx patients and should be evaluated in larger studies.

## Background

Lung transplantation (LTx) is an established therapeutic option to increase survival rates and improve quality of life in selected end-stage lung disease patients [1]. Despite increased survival rates, lung transplant recipients still have a poor prognosis compared with other organ transplant patients [1]. Long-term survival is limited by the occurrence of chronic lung allograft dysfunction (CLAD), an umbrella term used to describe various pathophysiological processes affecting the allograft [2], among them bronchiolitis obliterans syndrome (BOS), the most common form of CLAD, and less frequently restrictive allograft syndrome (RAS), and/or a combination of the two [3]. As the detection of BOS enables immediate therapeutic intervention, early diagnosis is essential.

The main clinical finding of BOS is an irreversible airway obstruction with a persistent (≥3 weeks) reduction of forced expiratory volume in 1 s (FEV_1_) [4], without evidence of acute infection, acute cellular and/or antibody-mediated rejection and other factors [5, 6]. The classification covers BOS-0 (no BOS), BOS-0-p (potential BOS), and BOS 1–3, based on increasing airway obstruction (FEV_1_) compared with the best-ever value post-transplantation. Moreover, a reduction in the mean forced expiratory flow during the middle half of forced vital capacity (FEF_25–75_) is used as an early marker [4, 7]. FEV_1_ is an easily measurable but non-specific parameter, which is unlikely to fully reflect the situation in the lung periphery, where BOS originates [8]. The fact that BOS is characterized by an abnormal remodelling of terminal bronchioles, leading to airflow limitation of the small airways [9], raises the question whether other lung parameters could provide more detailed information. It is known that total lung capacity (TLC) and the ratio of TLC and residual volume (RV) are predictors of CLAD and can help to identify high-risk patients [10]. Prior investigations from our group demonstrated that elevated levels of exhaled nitric oxide (FeNO) could predict the development of BOS in LTx patients and also could be an early marker of deterioration in patients with or without pre-existing functional impairment [11]. The data also showed that a marked increase in follow-up FeNO measurements could identify patients at risk for an unfavourable course, whereas stable LTx recipients showed a significantly lower individual variation in FeNO values [11]. Cameli et al. [12] investigated the role of exhaled NO (eNO) and carbon monoxide (eCO) as markers of pulmonary inflammation associated with acute graft rejection and lung infection in LTx patients and found higher values of FeNO and in particular a higher alveolar concentration of nitric oxide (Calv_NO_) in LTx patients with BOS compared to non-BOS patients [12]. This indicates that alveolar markers might be suitable for detecting BOS, as airway inflammation and remodelling of the small airways in LTx patients with BOS could be associated with alveolar changes such as elevated levels of peripheral eNO and Calv_NO_ [12]. Thus, gas-exchange parameters are also candidates for detecting the malfunction of peripheral structural changes. For this purpose the differentiation from a capillary disorder would be helpful, and this task could be performed by the diffusing capacity for nitric oxide (DLNO), especially in combination with that for carbon monoxide (DLCO). This combination has been evaluated in various lung diseases but is not yet part of clinical routine [13, 14].

The comparison of DLNO and DLCO allows for the differentiation of lung components affected by the lung disease, since the affinity for NO to hemoglobin is much higher compared with that of CO [15]. Therefore, NO uptake is largely unaffected by pulmonary capillary blood volume (Vcap), in contrast with CO [16, 17], while the diffusion resistance (membrane factor, DMCO) is similar for both gases. DLNO can therefore be used to assess the membrane factor, and in combination with DLCO the Vcap. This allows for the differentiation between a thickened alveolar capillary membrane and reduced blood flow which is not possible by DLCO alone [18]. In case of membrane thickening both DLCO and DLNO would be affected, but DLNO stronger, while in case of a reduced alveolar volume (V_A_) or a reduced Vcap, DLCO would decrease more than DLNO [19]. Although BOS has an obstructive component, it could also be associated with a transport disorder of the alveolar membrane.

Based on these considerations, the aim of this investigation was to reveal whether the non-invasive measurement of DLNO and DLCO is capable of an early detection of BOS in LTx recipients.

## Methods

### Study population

This cross-sectional study was performed at the Department of Internal Medicine V and the Institute and Outpatient Clinic for Occupational, Social and Environmental Medicine at the Ludwig Maximilian University of Munich, Germany. The cohort was recruited through the outpatient and inpatient sectors. A patient was enrolled in the cohort if he or she fulfilled the following inclusion criteria: (a) the patient was the recipient of a LTx performed at the study center, and (b) the patient expressed his or her willingness to participate in individually planned follow-up assessments; and if the patient did not fulfill any of the following exclusion criteria: (a) acute infections, (b) high levels of physical exertion or food intake prior to the measurements, (c) nicotine abuse on the day of measurements, or (d) physical impairment resulting in an inability to perform two valid and reproducible DLNO-DLCO measurements [13]. All assessments were approved by the local ethics committee (Ludwig Maximilian University of Munich, Germany) and all patients gave their written informed consent.

### Assessments

Patients underwent a panel of assessments in order to collect clinical characteristics and functional status. The following parameters were recorded: height, weight, date of birth, lung disease which led to LTx, date of transplantation, type of transplantation (single LTx (SLTx), bilateral LTx (BLTx)), BOS stage, allergies, past medication, date of last food intake, acute respiratory infections, and smoking history.

### Measurements combined diffusing capacity (DLNO-DLCO)

Simultaneous measurements of DLNO, DLCO and V_A_ were performed using the single-breath method (MasterScreen™ PFT Pro, Jaeger, CareFusion, Hoechberg, Germany). Although the study was performed prior to the recommendations of the American Thoracic Society and European Respiratory Society, [20] the study was in accordance with their standards. After careful instruction, patients performed three measurements at a breath-hold time of 8 s [21, 22] with at least five-minute resting intervals in between maneuvers to assure elimination of the test gas from the lungs. Incorrect measurements were repeated, to gain at least two plausible measurements, the mean of which was included in the further analyses. Using a breath-hold time of 8 s, excellent test-retest reliability has been reported for combined DLNO-DLCO measurements in patients with cystic fibrosis [21]. The test gas comprised a mixture of 0.28% carbon monoxide (CO), 600 mg/m^3^ nitric oxide (NO) and 9.5% helium. Again, the measurements of V_A_, DLCO and DLNO satisfied the technical standards proposed by Zavorsky et al. [23], although the study was performed prior to publication of these recommendations. The transfer coefficients for nitric oxide (KNO) and carbon monoxide (KCO) were calculated by dividing DLNO and DLCO through V_A_, while the alveolar-capillary membrane diffusing capacity for carbon monoxide (DMCO) and Vcap were derived as proposed [13]. Percent predicted values for DLNO and DLCO were calculated according to Zavorsky et al. [23] based on previous work by Roughton et al. [24] and Guènard et al. [16], and DLCO was adjusted for hemoglobin [13, 17, 18].

In addition to DLNO and DLCO, conventional parameters of spirometry and bodyplethysmography were collected [25, 26]. For analysis the forced expiratory volume in 1 s (FEV_1_) and forced vital capacity (FVC) were used, as well as intrathoracic gas volume (ITGV), residual volume (RV), total lung capacity (TLC), airway resistance (Raw) and specific airway resistance (sRaw). Percent predicted values for spirometry and bodyplethysmography were taken from the Global Lung Initiative [27], or the European Coal and Steel Community (ECCS) [28], respectively. Furthermore, routine laboratory parameters including hemoglobin, sodium, potassium, creatinine and CRP values were collected.

### Statistical analysis

For data description, mean values and standard deviations (SD) were used. Comparisons between groups were performed using non-parametric tests, especially the Mann-Whitney-U test and the Kruskal-Wallis test, as well as chi-squared statistics for categorical data. To evaluate the value of the combined diffusing capacity for BOS recognition, patients were grouped into binary categories according to their BOS-stage. For the purpose of early diagnosis (“BOS binary-early”), stages 0 and 0-p were combined and compared with stages 1–3. To reveal whether it was possible to differentiate an initial diagnosis from progression, stages 0, 0-p and 1 were combined versus the combination of stages 2 and 3; this classification was termed “BOS-binary-late”. The relationship between BOS groups and predictors was analyzed by means of linear discriminant analysis in order to identify the best parameters to differentiate between the respective groups. We used an approach with forward selection. The results were checked by logistic regression analyses performed in an analogous manner. The level of statistical significance (alpha) was set at 0.05 for all analyses. Statistical analyses were performed with SPSS Statistics 23 (IBM Corp., Armonk, NY, USA).

## Results

### Patient population

Sixty-four patients were screened for the study. Three patients were excluded because they were not capable of performing valid diffusing capacity measurements. Therefore, 61 patients (29 males, 32 females; mean ± SD age 50.8 ± 13.7 years) were included in the analysis. BLTx had been performed in 19/23 male/female patients. SLTx and BLTx patients showed no significant differences in anthropometric characteristics and the time between LTx and measurements, while BLTx patients were younger (45.8 ± 13.0 years, *p* < 0.001) than SLTx patients (61.8 ± 7.5 years).

### BOS stages and pre-existing conditions

Idiopathic lung fibrosis (IPF) was the reason for transplantation in 22 patients. Following the official American Thoracic Society Statement, IPF was defined as chronic, progressive fibrosing interstitial pneumonia of unknown cause occurring primarily in adults, limited to the lungs, with radiological signs and/or histopathologic patterns consistent with usual interstitial pneumonia [29]. Further reasons for transplantation were COPD with emphysema in 16 patients (2 with alpha-1-antitrypsine deficiency), cystic fibrosis in 11 patients, three patients suffered from sarcoidosis, while the remaining 9 patients formed a heterogeneous group of diagnoses, including systemic lupus erythematosis, lymphangioleiomyomatosis, silicosis, Kartagener syndrome, or hypersensitivity pneumonitis alveolitis. There was no significant association between BOS-stages and the pre-existing lung disease.

### BOS stages and patients’ characteristics

The average interval between LTx and time of measurement was 4.3 ± 3.7 years. Nineteen patients were diagnosed as BOS-0, 29 patients as BOS 0-p, 7 as BOS 1, 2 as BOS 2, and 3 as BOS 3 (Table 1). There were no significant differences for age and BMI (Table 1) between BOS stages, when using the full classification or the binary groups. There was a significant difference in the number of years after LTx (*p* = 0.001), with the shortest period of time in BOS 0 and the longest in BOS 3There were no significant differences in the laboratory parameters hemoglobin, creatinine, CRP, sodium and potassium across the BOS-stages.

**Table 1:** BMI and age versus BOS stages

Parameter	BOS	*N*	Mean ± SD
Age (y)	0	19	47.4 ± 15.1
	0-p	29	50.8 ± 12.6
	1	7	60.4 ± 10.9
	2	3	46.7 ± 21.6
	3	3	53.7 ± 9.3
	Total	61	50.8 ± 13.7
BMI (kg/m^2^)	0	19	22.3 ± 3.9
	0-p	29	24.3 ± 4.7
	1	7	22.9 ± 3.9
	2	3	23.9 ± 3.3
	3	3	21.5 ± 7.6
	Total	61	23.4 ± 4.4

The table shows mean values and standard deviations for age and body mass index (BMI) across the different BOS stages

### BOS stages, lung function and combined DLNO-DLCO

**Fig. 1:**
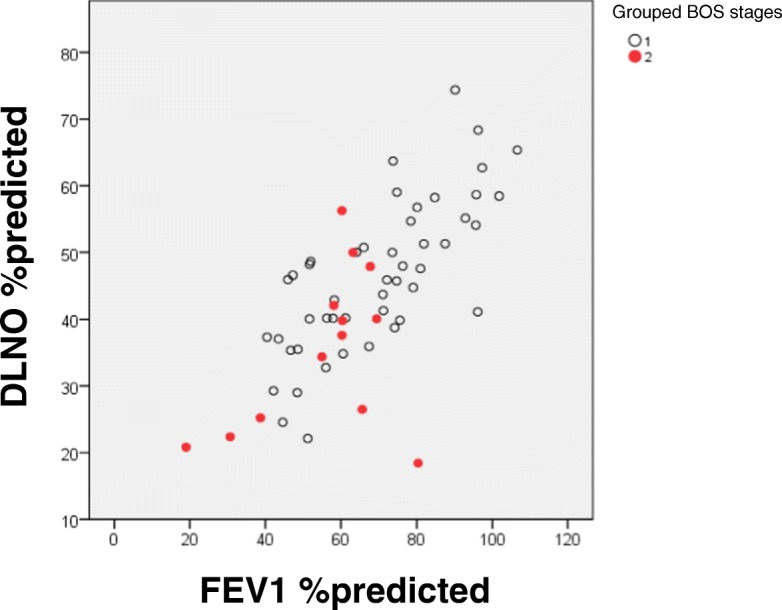
Detection of early BOS. Discrimination capability of DLNO %predicted versus FEV1%predicted in the detection of early BOS. 1 = BOS 0/0-p, 2 = BOS 1/2/3

Pulmonary function characteristics for all patients (*n* = 61) are shown in Tables 2 and 3. Increasing BOS stages were significantly associated with higher values of Raw and sRAw, and with lower values of FEV_1_ and FEV_1_/FVC (*p* < 0.05 each); RV/TLC and ITGV/TLC were borderline to non-significant (*p* = 0.055 each). Higher BOS stages were also significantly linked to lower values of DLCO, DLNO, and DMCO (p < 0.05 each). The discrimination capability of DLNO % predicted versus FEV_1_% predicted is shown in Fig. 1.

**Table 2:** Parameters of spirometry and bodyplethysmography versus BOS stages

	BOS	*N*	Mean ± SD	*p*-value
FEV_1_% predicted	0	19	73.1 ± 20.3	0.004
	0-p	29	67.4 ± 16.8	
	1	7	64.9 ± 8.2	
	2	3	62.0 ± 5.0	
	3	3	29.5 ± 9.9	
FVC % predicted	0	19	74.7 ± 18.0	0.045
	0-p	29	77.9 ± 17.3	
	1	7	72.2 ± 15.4	
	2	3	84.6 ± 12.2	
	3	3	46.6 ± 10.2	
FEV_1_/FVC % predicted	0	19	97.9 ± 15.3	0.002
	0-p	29	86.9 ± 14.8	
	1	7	92.6 ± 20.3	
	2	3	73.7 ± 11.5	
	3	3	62.2 ± 11.7	
TLC % predicted	0	19	88.4 ± 21.8	0.576
	0-p	29	110.1 ± 18.4	
	1	7	82.2 ± 30.5	
	2	3	107.2 ± 26.9	
	3	3	82.7 ± 3.1	
ITGV % predicted	0	19	102.9 ± 35.0	0.140
	0-p	29	100.1 ± 31.7	
	1	7	89.8 ± 39.3	
	2	3	146.4 ± 52.5	
	3	3	124.3 ± 15.4	
RV % predicted	0	19	108.8 ± 49.1	0.233
	0-p	29	119.9 ± 54.7	
	1	7	139.7 ± 83.0	
	2	3	75.7 ± 31.3	
	3	3	65.7 ± 12.6	
RV/TLC % predicted	0	19	118.1 ± 31.5	0.014
	0-p	29	107.9 ± 33.1	
	1	7	106.9 ± 27.0	
	2	3	131.9 ± 29.8	
	3	3	175.0 ± 24.4	
Raw (kPa*s*l^−1^)	0	19	0.27 ± 0.11	< 0.001
	0-p	29	0.31 ± 0.10	
	1	7	0.27 ± 0.09	
	2	3	0.39 ± 0.11	
	3	3	0.80 ± 0.19	
sRaw (kPa*s)	0	19	0.99 ± 0.65	< 0.001
	0-p	29	1.12 ± 0.52	
	1	7	0.84 ± 0.36	
	2	3	1.85 ± 1.06	
	3	3	3.56 ± 1.17	

The table shows mean values and standard deviations. The comparisons between groups were performed by ANOVA. *FEV*_*1*_ forced expiratory volume in one second, *FVC* forced vital capacity, *ITGV* intrathoracic gas volume, *TLC* total lung capacity, *RV* residual volume, *Raw*, airway resistance, *sRaw* specific airway resistance

**Table 3:** Parameters of diffusing capacity versus BOS stages

	BOS	*N*	Mean ± SD	*p*-value
DLCO % predicted	0	19	52.0 ± 13.8	0.018
	0-p	29	58.0 ± 12.5	
	1	7	44.3 ± 12.1	
	2	3	63.8 ± 18.3	
	3	3	37.9 ± 14.9	
KCO % predicted	0	19	75.8 ± 13.2	0.439
	0-p	29	84.1 ± 15.0	
	1	7	77.7 ± 13.7	
	2	3	87.0 ± 38.9	
	3	3	77.3 ± 25.8	
DLNO % predicted	0	19	44.1 ± 11.8	0.002
	0-p	29	47.9 ± 11.0	
	1	7	35.3 ± 10.2	
	2	3	48.8 ± 7.1	
	3	3	22.8 ± 2.2	
KNO % predicted	0	19	66.0 ± 11.1	0.014
	0-p	29	70.5 ± 9.1	
	1	7	62.9 ± 12.4	
	2	3	65.7 ± 19.2	
	3	3	48.4 ± 4.2	
DMCO % predicted	0	19	34.0 ± 10.6	0.004
	0-p	29	37.3 ± 11.0	
	1	7	27.4 ± 9.5	
	2	3	39.6 ± 5.6	
	3	3	14.7 ± 0.7	
V_A_ % predicted	0	19	67.5 ± 14.7	0.056
	0-p	29	67.4 ± 14.1	
	1	7	55.7 ± 16.7	
	2	3	71.2 ± 13.0	
	3	3	46.5 ± 6.4	
Vcap % predicted	0	19	69.2 ± 18.9	0.290
	0-p	29	75.0 ± 21.4	
	1	7	58.2 ± 14.5	
	2	3	75.6 ± 22.8	
	3	3	93.9 ± 82.6	
Vcap/V_A_ % predicted	0	19	106.7 ± 25.6	0.031
	0-p	29	116.5 ± 36.5	
	1	7	111.1 ± 29.4	
	2	3	115.4 ± 56.5	
	3	3	201.9 ± 167.0	
DMCO/V_A_ % predicted	0	19	52.8 ± 13.1	0.044
	0-p	29	57.3 ± 12.0	
	1	7	51.9 ± 14.7	
	2	3	59.3 ± 21.7	
	3	3	33.1 ± 3.1	

The table shows mean values and standard deviations. The comparisons between groups were performed by ANOVA. *DLCO* diffusing capacity for carbon monoxide, *DMCO* alveolar-capillary membrane diffusing capacity for carbon monoxide, *DLNO* diffusing capacity for nitric oxide, *KCO* transfer factor for carbon monoxide, *KNO* transfer factor for nitric oxide; *V*_*A*_ alveolar volume, *Vcap* pulmonary capillary blood volume

### Recognition of early BOS

**Fig. 2:**
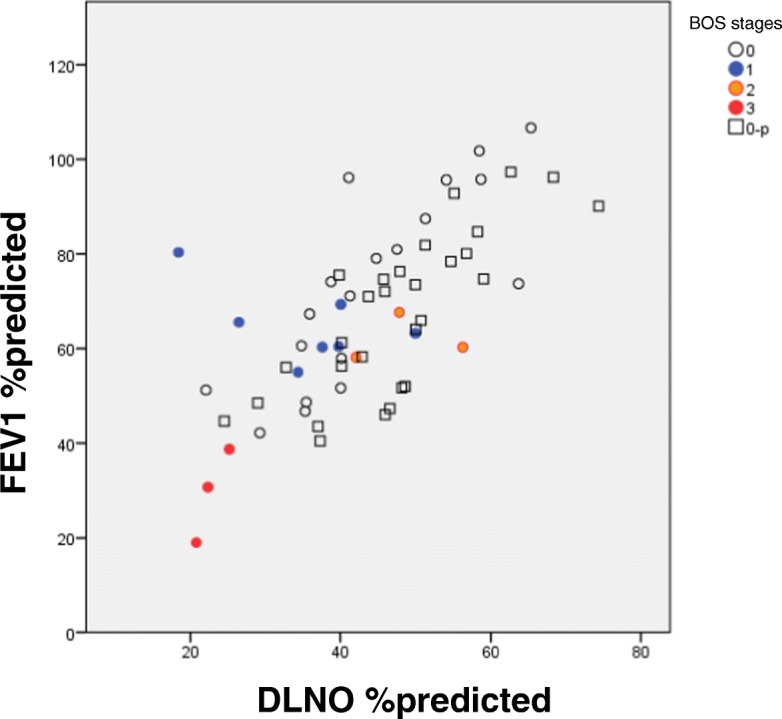
Detection of BOS. Discrimination capability of DLNO %predicted versus FEV1%predicted. In this figure the five different BOS categories are indicated separately

The comparison of pulmonary function parameters between the two groups BOS 0/0-p and BOS 1–3 (BOS binary-early) showed lower values of FEV_1_ and higher values of RV/TLC (Mann-Whitney-U test, *p* < 0.05 each). When comparing diffusing capacity parameters between groups, DLCO, DLNO, V_A_ and DMCO turned out to be significantly reduced in the BOS 1–3 group, either regarding the absolute or % predicted values (Mann-Whitney-U test, *p* < 0.05). The individual data for FEV_1_ and DLNO are shown in Fig. 2.

In a discriminant analysis using the spirometric parameters only, FEV_1_% predicted was identified as the major variable separating the two BOS groups “BOS binary-early” (specificity 60.4%, sensitivity 61.5%, positive predictive value 60.7%). It should be noted that the numbers of patients in both groups were unbalanced (*n* = 48 versus *n* = 13), and that sensitivity and specificity were low. Likewise, when performing the analysis with bodyplethysmographic parameters only, Raw was the only significant variable discriminating between the two BOS groups (specificity 70.8%; sensitivity 46.2%, positive predictive value 65.5%). When repeating the discriminant analysis with data from both spirometry and bodyplethysmography, Raw again remained as major variable separating the two groups “BOS binary-early”, thus the combination of data had no additive value.

When the analysis was performed with all directly measured and derived parameters of the combined diffusing capacity, DLNO % predicted remained as only significant variable separating the two groups “BOS binary-early” (specificity 64.6%, sensitivity 69.2%, positive predictive value 65.6%). Since the combined measurement allows for the separation of membrane factor and Vcap, in a separate analysis only the derived parameters were tested, which revealed the membrane factor DMCO % predicted as only significant variable for “BOS binary-early” (positive predictive value 60.7%); Vcap played no role. As DMCO depends on DLNO, this result is plausible.

Finally, to compare the predictive value between the three lung function domains described above, a discriminant analysis with the best parameters from each; i.e., FEV_1_, DLNO or DMCO (each in % predicted), and Raw was performed. In this analysis DLNO % predicted was the dominant and only significant variable discriminating between no BOS and “BOS binary-early” (positive predictive value as above: 65.6%). This finding demonstrated that DLNO carried more information than spirometry and bodyplethysmography in the detection of early BOS stages, even though the specificity was low. All of the above results were confirmed by logistic regression analysis.

### Recognition of advanced BOS stages

**Fig. 3:**
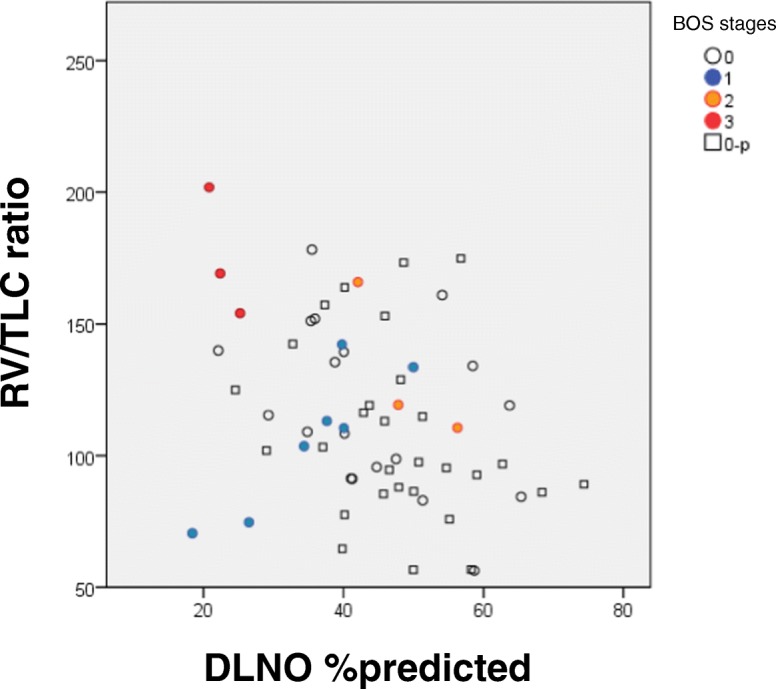
Detection of BOS. Discrimination capability of RV/TLC ratio versus DLNO % predicted. In this figure the five different BOS categories are indicated separately

To analyze advanced BOS stages, patients were grouped into BOS 0–1 versus BOS 2–3, although this grouping led to an even greater imbalance of group sizes (*n* = 55 versus *n* = 6). In a discriminant analysis using only spirometric parameters, most of them were significant in separating the two groups “BOS binary-late”. FEV_1_ and FEV_1_/FVC, both in % predicted, achieved the strongest separation, whereas FVC % predicted was not significant. The positive predictive value of 82.0%, with high specificity (85.5%) but low sensitivity (50.0%) indicated a bias due to the unbalanced group sizes. When performing the analysis with bodyplethysmographic parameters only, all parameters except for TLC % predicted were significant in discriminating between the two BOS groups, with Raw and sRaw as most informative parameters (*p* < 0.001 each; sRaw: specificity 90.9%, sensitivity 66.7%, positive predictive value 88.5%). To evaluate the role of the other bodyplethysmographic parameters, the analysis was repeated without Raw and sRaw. ITGV/TLC % predicted was the only remaining significant variable (specificity 76.4%, sensitivity 66.7%, positive predictive value 75.4%), while the RV/TLC ratio was inferior to this. The discrimination capability of RV/TLC ratio versus DLNO % predicted is shown in Fig. 3. The discriminant analysis using only parameters of diffusing capacity except DLNO/DLCO ratio and DMCO/Vcap showed that KNO, KCO and DMCO/VA, each in % predicted, were significant in separating the two BOS groups. The addition of the DLNO/DLCO ratio and DMCO/Vcap in the discriminant analysis revealed DLNO/DLCO ratio as only remaining variable significantly separating the “BOS binary-late” groups (specificity 74.5%, sensitivity 66.7%, positive predictive value 73.8%). The results given above were confirmed by logistic regression analysis.

## Discussion

This study revealed that the diffusing capacity of the lung for NO allows the early detection of BOS in patients following BLTx but has no additional value for patients following SLTx.. Compared to a panel of conventional lung function measures DLNO was the parameter with the highest reliability in the detection of early BOS stages. By contrast, we were not able to show an additional value of DLNO for the detection of advanced BOS stages within the limits of a very small number of patients with advanced BOS stages.

The study population was comprised of 61 patients with well-balanced gender distribution, and the proportion of SLTx and BLTx patients (*n* = 19 versus 42) was in accordance with the international practice favoring BLTx [1]. The only statistically significant difference between SLTx and BLTx patients was age, due to the fact that SLTx was mainly performed in patients with emphysema, whereas BLTx was mostly chosen in younger patients with cystic fibrosis, in line with international data [1, 30]. Thus, the study population can be regarded as representative.

Regarding the early diagnosis of BOS, discriminant analyses identified FEV_1_% predicted, Raw and DLNO % predicted as the parameters from spirometry, bodyplethysmography and combined NO-CO diffusing capacity, respectively, which could best differentiate between both groups. In the competition of these parameters, DLNO % predicted outperformed the other two parameters which yielded no additional information. Therefore, in line with our hypothesis, DLNO carried substantial information regarding the detection of early BOS stages. The major pathophysiological changes leading to BOS include inflammation, repetitive injury of airway epithelia, fibrotic changes and airway remodelling [9]; thus, it is reasonable to expect an impact on pulmonary function due to the resulting membrane thickening, which should be reflected in changes of DLNO. BOS histopathology is in the early stages characterized by inflammation and fibrosis of the airway with sparing of the surrounding alveoli. Still, this raises the possibility that the gas transport to and within the alveoli is affected, which could be detected by a highly sensitive method assessing gas uptake without major interference with factors arising from the pulmonary capillaries. In our study we tested whether DLNO could achieve this task, as a sort of indirect test of peripheral airway dysfunction.

In diagnosing advanced BOS stages the results were different, and FEV_1_ and FEV_1_/FVC, each in % predicted, emerged as most informative parameters. This is reasonable insofar as FEV_1_ is used for the definition of these BOS stages. From bodyplethysmography, sRaw and ITGV/TLC were informative, while in the combined diffusing capacity the DLNO/DLCO ratio remained as sole differentiating variable. When comparing these parameters to determine their relative predictive value, FEV_1_/VC and ITGV/TLC remained informative, while the combined DLNO-DLCO carried no additional value. To facilitate the comparison with the literature [10], instead of ITGV/TLC the ratio RV/TLC was also tested, leading to similar results.

In the evaluation of all results, the inhomogeneous group sizes must be considered. The group “BOS-binary-late” was comprised of 6 versus 55 patients, whereas in the group “BOS-binary-early” the relationship was more favourable with 13 versus 48 patients. Although the analysis of the advanced stages was heavily handicapped by the unbalanced group sizes, the results are consistent with known data regarding RV/TLC [10], possibly indicating that the relative unimportance of DLNO in this condition was a valid result.

Thus far, the combined DLNO-DLCO has not been investigated in patients post LTx for the detection of BOS. Regarding the early diagnosis DLNO was superior to all other lung function parameters under study, confirming our hypothesis that DLNO is especially sensitive to changes in peripheral airways which are common in early stages of BOS. This is remarkable because the definition of BOS is based not on single measurements but on the individual time course of spirometric measurements, while we performed only single measurements expressed as % predicted. The fact that DLNO was superior even to FEV_1_ used in the definition of BOS is a hint that the sensitivity of DLNO could be even greater when used in individual follow-up measurements. In eight patients, repeated measurements were available at an average time interval of 99 days, demonstrating good reproducibility, but no incident BOS cases occurred during this time period.

As expected, FEV_1_ showed a significant capability of discrimination between BOS stages, as well as RV/TLC, Raw and sRaw, but only if the combined DLNO-DLCO was not included. Former investigations revealed that a high RV/TLC ratio, as indicator of hyperinflation and air trapping, predicts survival in patients with CLAD [10]. In our study the RV/TLC ratio was also significantly higher in patients with BOS 2–3 as well as in BOS 1–3, compared to the other BOS stages. Despite this, the ratio was inferior to DLNO in the detection of early BOS. Regarding advanced BOS stages the results for RV/TLC were in line with a previous publication from our group [10].

The measurement of DLNO-DLCO was performed before the publication of the European Respiratory Society (ERS) technical standards document for the single-breath CO and NO diffusing capacity [20, 13]. Therefore, the procedure of measurements followed previous approaches published by van der Lee et al. [31] and Dressel et al. [21]. Irrespective of this, the measurements were in accordance with recent recommendations for single-breath DLCO [20, 32] as well as DLNO [13], taking into account that breath-hold time was within the range, in which diffusing capacity is not markedly affected by different breath-hold times [33].

The combined DLNO-DLCO allows the non-invasive discrimination between alterations of gas uptake resulting from lung parenchyma versus pulmonary capillary circulation. This capability has proved useful in different diseases such as heart failure [34], liver cirrhosis [35], pulmonary arterial hypertension [17, 36], chronic obstructive pulmonary disease [37], cystic fibrosis [22] and stem cell transplantation [38]. In the present study, we could extend this range to lung transplant recipients. Apparently, early structural and/or inflammatory changes in the lung of patients with BOS deteriorate alveolar gas uptake as measurable by DLNO, whereas DLCO, being more dependent on Vcap, seems less affected.

Additional analyses revealed that DLNO had no value in recognizing “early BOS” in single-lung transplant patients, whereas it still showed a significant difference in double-lung transplant patients (Mann-Whitney-U test, *p* = 0.002). This might indicate differences arising from the different clinical background which led to either single or double transplantation. Even more likely is that in single-lung transplant patients the remaining lung still affected from the background disease dominates the test results thereby preventing the use of DLNO.

### Limitations and strengths

The major limitation of our analysis was the small study cohort; in particular, the low number of patients with advanced BOS stages. Moreover, the sample size was not sufficient to find out whether DLNO is even of value in discriminating between BOS 0 and 0-p; individual follow-up measurements may be necessary for this. Nevertheless, at least the result for the early detection of BOS appears plausible enough to motivate larger, multi-center follow-up studies. This appears feasible, as the measurement of the combined diffusing capacity is standardized, can be performed simultaneously with the DLCO measurement and does not require specific cooperation or expertise. In light of the poor prognosis of LTx patients, any additional non-invasive parameter that could help in disease monitoring is highly appreciated. As BOS causes changes in the lung periphery, parameters targeting this part of the lung are particularly promising, and among these DLNO appears to be a prime candidate, at least in patients with double-lung transplants.

## Abbreviations

ANOVA: Analysis of variance
BLTx: Bilateral LTx
BOS: Bronchiolitis obliterans syndrome
CLAD: Chronic lung allograft dysfunction
CO: Carbon monoxide
DLCO: Carbon monoxide
DLNO: Diffusing capacity for nitric oxide
DMCO: Diffusion resistance, membrane factor
FEF_25–75_: Middle half of forced vital capacity
FEV_1_: Forced expiratory volume in 1 s
FVC: Forced vital capacity
ITGV: Intrathoracic gas volume
KCO: Transfer coefficients for carbon monoxide
KNO: Transfer coefficients for nitric oxide
LTx: Lung transplantation
NO: Nitric oxide
RAS: Restrictive allograft syndrome
Raw: Airway resistance
RV: Residual volume
SD: Standard deviations
SLTx: Single LTx
sRaw: Specific airway resistance
TLC: Total lung capacity
V_A_: Alveolar volume
Vcap: Pulmonary capillary blood volume

## References

[1] Chambers DC, Yusen RD, Cherikh WS, Goldfarb SB, Kucheryavaya AY, Khusch K, Levvey BJ, Lund LH, Meiser B, Rossano JW (2017). The Registry of the International Society for Heart and Lung Transplantation: Thirty-fourth Adult Lung And Heart-Lung Transplantation Report-2017; Focus Theme: Allograft ischemic time. J Heart Lung Transplant.

[2] Yusen RD, Edwards LB, Kucheryavaya AY, Benden C, Dipchand AI, Goldfarb SB, Levvey BJ, Lund LH, Meiser B, Rossano JW (2015). The Registry of the International Society for Heart and Lung Transplantation: Thirty-second Official Adult Lung and Heart-Lung Transplantation Report--2015; Focus Theme: Early Graft Failure. J Heart Lung Transplant.

[3] Royer PJ, Olivera-Botello G, Koutsokera A, Aubert JD, Bernasconi E, Tissot A, Pison C, Nicod L, Boissel JP, Magnan A (2016). Chronic Lung Allograft Dysfunction: A Systematic Review of Mechanisms. Transplantation.

[4] Hayes D (2011). A review of bronchiolitis obliterans syndrome and therapeutic strategies. J Cardiothorac Surg.

[5] Levine DJ, Glanville AR, Aboyoun C, Belperio J, Benden C, Berry GJ, Hachem R, Hayes D, Jr., Neil D, Reinsmoen NL et al.: Antibody-mediated rejection of the lung: A consensus report of the International Society for Heart and Lung Transplantation. J Heart Lung Transplant 2016, 35(4):397–406. 10.1016/j.healun.2016.01.1223 27044531

[6] Sato M, Waddell TK, Wagnetz U, Roberts HC, Hwang DM, Haroon A, Wagnetz D, Chaparro C, Singer LG, Hutcheon MA (2011). Restrictive allograft syndrome (RAS): a novel form of chronic lung allograft dysfunction. J Heart Lung Transplant.

[7] Estenne M, Maurer JR, Boehler A, Egan JJ, Frost A, Hertz M, Mallory GB, Snell GI, Yousem S (2002). Bronchiolitis obliterans syndrome 2001: an update of the diagnostic criteria. J Heart Lung Transplant.

[8] Yousem SA, Duncan SR, Griffith BP (1992). Interstitial and airspace granulation tissue reactions in lung transplant recipients. Am J Surg Pathol.

[9] Verleden SE, Sacreas A, Vos R, Vanaudenaerde BM, Verleden GM (2016). Advances in Understanding Bronchiolitis Obliterans After Lung Transplantation. Chest.

[10] Kneidinger N, Milger K, Janitza S, Ceelen F, Leuschner G, Dinkel J, Konigshoff M, Weig T, Schramm R, Winter H et al.: Lung volumes predict survival in patients with chronic lung allograft dysfunction. Eur Respir J 2017, 49(4). 10.1183/13993003.01315-2016 28404648

[11] Neurohr C, Huppmann P, Leuschner S, von Wulffen W, Meis T, Leuchte H, Ihle F, Zimmermann G, Baezner C, Hatz R (2011). Usefulness of exhaled nitric oxide to guide risk stratification for bronchiolitis obliterans syndrome after lung transplantation. Am J Transplant.

[12] Cameli P, Bargagli E, Fossi A, Bennett D, Voltolini L, Refini RM, Gotti G, Rottoli P (2015). Exhaled nitric oxide and carbon monoxide in lung transplanted patients. Respir Med.

[13] Zavorsky GS, Hsia CC, Hughes JM, Borland CD, Guenard H, van der Lee I, Steenbruggen I, Naeije R, Cao J, Dinh-Xuan AT. Standardisation and application of the single-breath determination of nitric oxide uptake in the lung. Eur Respir J. 2017:49(2). 10.1183/13993003.00962-2016 28179436

[14] Nyilas S, Carlens J, Price T, Singer F, Muller C, Hansen G, Warnecke G, Latzin P, Schwerk N (2018). Multiple breath washout in pediatric patients after lung transplantation. Am J Transplant.

[15] Gibson QH, Roughton FJ (1957). The kinetics and equilibria of the reactions of nitric oxide with sheep haemoglobin. J Physiol.

[16] Guenard H, Varene N, Vaida P (1987). Determination of lung capillary blood volume and membrane diffusing capacity in man by the measurements of NO and CO transfer. Respir Physiol.

[17] Borland C, Bottrill F, Jones A, Sparkes C, Vuylsteke A (2014). The significant blood resistance to lung nitric oxide transfer lies within the red cell. J Appl Physiol (1985).

[18] van der Lee I, Zanen P, Biesma DH, van den Bosch JM (2005). The effect of red cell transfusion on nitric oxide diffusing capacity. Respiration.

[19] Hughes JM, van der Lee I (2013). The TL,NO/TL,CO ratio in pulmonary function test interpretation. Eur Respir J.

[20] Graham BL, Brusasco V, Burgos F, Cooper BG, Jensen R, Kendrick A, MacIntyre NR, Thompson BR, Wanger J. Executive Summary: 2017 ERS/ATS standards for single-breath carbon monoxide uptake in the lung. Eur Respir J. 2017;49(1). 10.1183/13993003.00016-2016. 10.1183/13993003.00016-2016 28049168

[21] Dressel H, Filser L, Fischer R, de la Motte D, Steinhaeusser W, Huber RM, Nowak D, Jorres RA (2008). Lung diffusing capacity for nitric oxide and carbon monoxide: dependence on breath-hold time. Chest.

[22] Dressel H, Filser L, Fischer R, Marten K, Muller-Lisse U, de la Motte D, Nowak D, Huber RM, Jorres RA (2009). Lung diffusing capacity for nitric oxide and carbon monoxide in relation to morphological changes as assessed by computed tomography in patients with cystic fibrosis. BMC Pulm Med.

[23] Zavorsky GS (2017). Nitric oxide uptake in the lung: It is about time that clinicians use this test routinely. Respir Physiol Neurobiol.

[24] Roughton FJ, Forster RE (1957). Relative importance of diffusion and chemical reaction rates in determining rate of exchange of gases in the human lung, with special reference to true diffusing capacity of pulmonary membrane and volume of blood in the lung capillaries. J Appl Physiol.

[25] Criee CP, Sorichter S, Smith HJ, Kardos P, Merget R, Heise D, Berdel D, Kohler D, Magnussen H, Marek W (2011). Body plethysmography--its principles and clinical use. Respir Med.

[26] Miller MR, Hankinson J, Brusasco V, Burgos F, Casaburi R, Coates A, Crapo R, Enright P, van der Grinten CP, Gustafsson P et al: Standardisation of spirometry. Eur Respir J 2005, 26(2):319–338. 10.1183/09031936.05.00034805 16055882

[27] Quanjer PH, Stanojevic S, Cole TJ, Baur X, Hall GL, Culver BH, Enright PL, Hankinson JL, Ip MS, Zheng J (2012). Multi-ethnic reference values for spirometry for the 3–95-yr age range: the global lung function 2012 equations. Eur Respir J.

[28] Quanjer PH, Tammeling GJ, Cotes JE, Peslin R, Yernault JC, Pedersen OF (1993). Lung volumes and forced ventilatory flows. Eur Respir J.

[29] Raghu G, Collard HR, Egan JJ, Martinez FJ, Behr J, Brown KK, Colby TV, Cordier JF, Flaherty KR, Lasky JA (2011). An official ATS/ERS/JRS/ALAT statement: idiopathic pulmonary fibrosis: evidence-based guidelines for diagnosis and management. Am J Respir Crit Care Med.

[30] Schuba B, Scheklinski M, von Dossow V, Schneider C, Preissler G, Kneidinger N, Neurohr C, Michel S, Hagl C, Schramm R: Five-year experience using the Lung Allocation Score: the Munich Lung Transplant Group. Eur J Cardiothorac Surg 201854(2):328-333. 10.1093/ejcts/ezy035 29462335

[31] van der Lee I, van Es HW, Noordmans HJ, van den Bosch JM, Zanen P (2006). Alveolar volume determined by single-breath helium dilution correlates with the high-resolution computed tomography-derived nonemphysematous lung volume. Respiration.

[32] Stanojevic S, Graham BL, Cooper BG, Thompson BR, Carter KW, Francis RW, Hall GL. Global Lung Function Initiative Twg, Global Lung Function Initiative T: Official ERS technical standards: Global Lung Function Initiative reference values for the carbon monoxide transfer factor for Caucasians. Eur Respir J. 2017;50(3):139-43. 10.1183/13993003.00010-2017 28893868

[33] Radtke T, Benden C, Maggi-Beba M, Kriemler S, van der Lee I, Dressel H (2017). Intra-session and inter-session variability of nitric oxide pulmonary diffusing capacity in adults with cystic fibrosis. Respir Physiol Neurobiol.

[34] Magini A, Apostolo A, Salvioni E, Italiano G, Veglia F, Agostoni P (2015). Alveolar-capillary membrane diffusion measurement by nitric oxide inhalation in heart failure. Eur J Prev Cardiol.

[35] Degano B, Mittaine M, Guenard H, Rami J, Garcia G, Kamar N, Bureau C, Peron JM, Rostaing L, Riviere D (2009). Nitric oxide and carbon monoxide lung transfer in patients with advanced liver cirrhosis. J Appl Physiol (1985).

[36] Farha S, Laskowski D, George D, Park MM, Tang WH, Dweik RA, Erzurum SC (2013). Loss of alveolar membrane diffusing capacity and pulmonary capillary blood volume in pulmonary arterial hypertension. Respir Res.

[37] van der Lee I, Gietema HA, Zanen P, van Klaveren RJ, Prokop M, Lammers JW, van den Bosch JM (2009). Nitric oxide diffusing capacity versus spirometry in the early diagnosis of emphysema in smokers. Respir Med.

[38] Le Bourgeois A, Malard F, Chevallier P, Urbistandoy G, Guillaume T, Delaunay J, Peterlin P, Lemarchand P, Germaud P, Mohty M (2016). Impact of pre-transplant diffusion lung capacity for nitric oxide (DLNO) and of DLNO/pre-transplant diffusion lung capacity for carbon monoxide (DLNO/DLCO) ratio on pulmonary outcomes in adults receiving allogeneic stem cell transplantation for hematological diseases. Bone Marrow Transplant.

